# Digoxin-Mediated Inhibition of Potential Hypoxia-Related Angiogenic Repair in Modulated Electro-Hyperthermia (mEHT)-Treated Murine Triple-Negative Breast Cancer Model

**DOI:** 10.1021/acsptsci.3c00296

**Published:** 2024-01-09

**Authors:** Syeda Mahak Zahra Bokhari, Kenan Aloss, Pedro Henrique Leroy Viana, Csaba András Schvarcz, Balázs Besztercei, Nino Giunashvili, Dániel Bócsi, Zoltán Koós, Andrea Balogh, Zoltán Benyó, Péter Hamar

**Affiliations:** † Institute of Translational Medicine, Semmelweis University, Üllői út 26, Budapest 1085, Hungary; ‡ Cerebrovascular and Neurocognitive Disorders Research Group, Eötvös, Loránd Research Network and Semmelweis University (ELKH-SE), Tűzoltó utca 37-47, Budapest 1094, Hungary

**Keywords:** triple-negative breast cancer, modulated electro-hyperthermia, hypoxia-inducible factor 1-α, digoxin

## Abstract

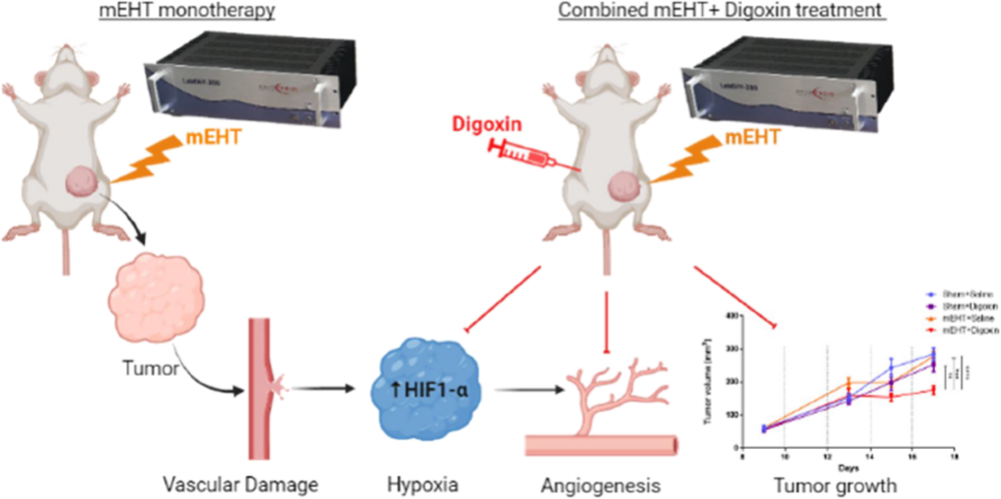

Triple-negative breast cancer (TNBC) is a highly aggressive breast cancer type with no targeted therapy and hence limited treatment options. Modulated electrohyperthermia (mEHT) is a novel complementary therapy where a 13.56 MHz radiofrequency current targets cancer cells selectively, inducing tumor damage by thermal and electromagnetic effects. We observed severe vascular damage in mEHT-treated tumors and investigated the potential synergism between mEHT and inhibition of tumor vasculature recovery in our TNBC mouse model. 4T1/4T07 isografts were orthotopically inoculated and treated three to five times with mEHT. mEHT induced vascular damage 4–12 h after treatment, leading to tissue hypoxia detected at 24 h. Hypoxia in treated tumors induced an angiogenic recovery 24 h after the last treatment. Administration of the cardiac glycoside digoxin with the potential hypoxia-inducible factor 1-α (HIF1-α) and angiogenesis inhibitory effects could synergistically augment mEHT-mediated tumor damage and reduce tissue hypoxia signaling and consequent vascular recovery in mEHT-treated TNBC tumors. Conclusively, repeated mEHT induced vascular damage and hypoxic stress in TNBC that promoted vascular recovery. Inhibiting this hypoxic stress signaling enhanced the effectiveness of mEHT and may potentially enhance other forms of cancer treatment.

Triple-negative breast cancer (TNBC) is an aggressive breast cancer subtype, characterized by a lack of expression of progesterone (PR), estrogen (ER), and human growth factor-receptor 2 (HER-2).^1^ TNBC accounts for 15–20% of all breast cancer types. In 2018, about 2 million women were diagnosed with TNBC which makes it a common cancer type.^2^ TNBC is a highly aggressive cancer with high metastatic potential and recurrence rates.^3^ Current treatment modalities include chemotherapies; however, there is no standard/specific treatment available.^4^ TNBC has poor prognosis with an average survival rate of 10.2 months with available treatments.^5^ New therapeutic modalities, such as (neo)adjuvant therapies, may help to manage TNBC.

Modulated electrohyperthermia (mEHT) is an efficient noninvasive adjuvant treatment with prominent clinical benefits.^6^ mEHT provides cytotoxic effects by loco-regional targeting of tumor tissue by amplitude-modulated (AM), 13.56 MHz frequency (radiofrequency) electromagnetic field.^7^ Mechanistically, mEHT benefits from the altered cell membrane profile and metabolism of tumor cells. Tumor cells owing to their altered bioelectric properties absorb more energy as compared to healthy surrounding cells, enabling mEHT to induce specific tumor damage and tumor cell death.^8,9^

Tumor vasculature is an important and complex factor in the tumor cell survival and proliferation. The abnormal morphology of tumor vasculature hinders blood perfusion and hence reduces the oxygen supply in the tumor core, promoting a hypoxic tumor microenvironment.^10^ This hypoxic microenvironment promotes angiogenesis to ensure tumor survival and proliferation.^11^ Although inhibition of angiogenic pathways has provided better patient outcomes in clinical settings, resistance to antiangiogenic therapy^12^ and tumor cells switching to other neovascularization processes are prominent challenges.^13^ In the present study, we investigated tumor hypoxia with pimonidazole staining. Pimonidazole is a nitroimidazole which specifically undergoes reduction and binds the thiol groups of proteins and amides in hypoxic cells, resulting in the formation of adducts which can be detected by an antipimonidazole antibody.^14^ Tumor vasculature was assessed by the mRNA expression of CD105 (endoglin), a marker for tumor vasculature in proliferating solid tumors,^15^ and the blood vasculature endothelial marker CD-31 staining.

We hypothesized that targeting hypoxia signaling in the tumor tissue may hinder neovascularization in our TNBC mouse model. Digoxin, a cardiac glycoside, is a Na/K ATPase inhibitor^16^ and a common drug of choice for patients with arrhythmias and congestive heart failure. Over the years, its potential as an anticancer therapy has been investigated.^17−19^ There are several clinical trials running to assess the ability of digoxin as a treatment modality for cancer.^20−29^ Zhang et al. reported that digoxin potently inhibited HIF1-α translation and reduced tumor volume.^18^ Digoxin further inhibited angiogenesis and tumor volume effectively in various cancer types.^30−32^ The effectiveness of digoxin in combination with chemotherapies such as doxorubicin,^33^ adriamycin,^32^ and gemcitabine^34^ have been already investigated.

In this paper, we demonstrate the effects of repeated mEHT treatments on tumor vasculature in TNBC models. Our study provides time-related insights into the vascular damage elicited by mEHT and a consequent tissue hypoxia. We observed vascular recovery following the time point of maximal hypoxia, suggesting an angiogenic repair initiated in response to hypoxia. Furthermore, we could limit the vascular repair by inhibiting tumor hypoxia signaling by digoxin in combination with mEHT. Thus, our findings suggest that mEHT and digoxin have a synergistic antitumor efficacy. Therefore, both mEHT and digoxin can potentiate each other’s cytotoxic ability in tumor cells, enabling a more effective therapy.

## 1 Results

### 1.1 mEHT Induced Interstitial Blood Leakage and Capillary Damage in TNBC Isografts

**Figure 1:**
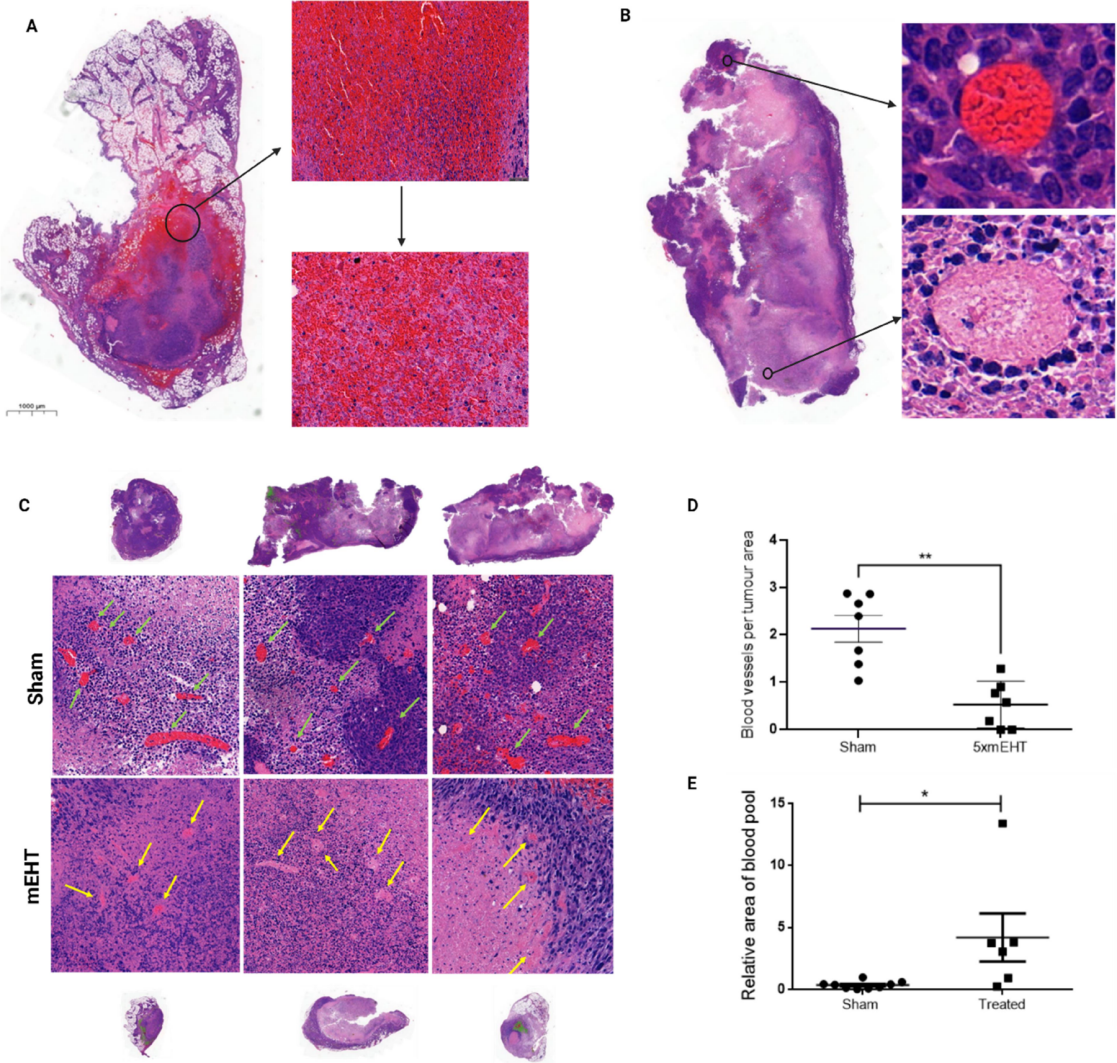
Capillary damage and interstitial blood in TNBC tumors after five mEHT treatments. (A) Large blood-covered area in a 4T1 TNBC tumor (H&E staining: 0.9×), erythrocytes between tumor cells in the interstitium (20×, 40×). (B) Representative images of red blood cell-filled capillary in a living tumor area and a dead capillary in the necrotic tumor area. The capillary is surrounded by dead tumor cells and mononuclear cells (H&E, 0.9×, 40×). (C) Representative images of tumor samples (H&E, 0.9×, 15×), (Pale areas = necrotic, dark areas = living tissue). Green arrows indicate intact capillaries, and yellow arrows point at destroyed capillaries. (D) Number of functional capillaries. (E) Blood-covered area relative to the total tumor area; unpaired Mann–Whitney test. Mean ± SEM, *n* = 6–8/group, **p = 0.0012, *p < 0.05. H&E (0.9×, 20×)

**Figure 2:**
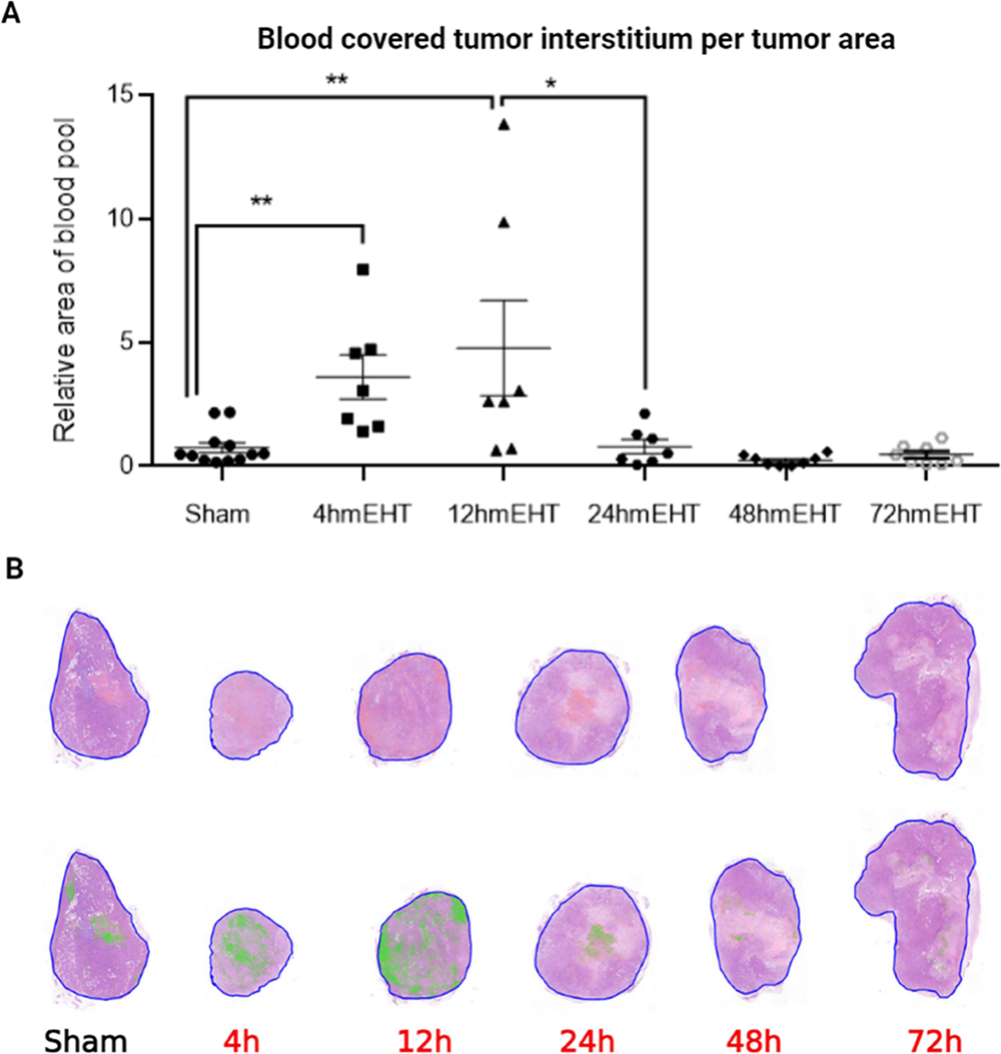
Kinetics of interstitial bleeding after three mEHT treatments. (A) Quantification of relative blood-covered area on H&E-stained tumors (1×). (B) Representative images of H&E-stained tumor samples with RBC-covered areas masked green within the blue annotated area; unpaired Mann–Whitney test. Mean ± SEM, *n* = 6–12/group, *p < 0.05, **p = 0.0012, **p = 0.0026.

Untreated tumors had an organized capillary structure, without observable blood in the interstitium. After five mEHT treatments, a striking observation was large red areas on H&E-stained sections. The cause of the red color was the accumulation of red blood cells (RBCs) as identified by high magnification (Figure 1A). In the same tumors, functional capillaries were characterized as filled with red blood cells in the living areas of the tumor tissue or surrounded by living cells distinct from the dead blood capillaries in damaged tumor areas (Figure 1B). The functional capillaries were significantly reduced in mEHT-treated tumors as compared to sham-treated tumors (Figure 1C,D). The reduced number of capillaries was accompanied by larger RBC-covered areas. Upon quantification, the tumor area covered by RBCs was significantly higher in mEHT-treated tumors as compared to the sham-treated ones (Figure 1E). Similarly, interstitial blood pools were observed 24 h after three mEHT treatments in a time-dependent manner. Four hours post treatment, a significant increase in interstitial blood was observed. The interstitial blood-covered area peaked at 12 h and was significantly reduced at 24 h and was further reduced to the sham level by 48–72 h after the mEHT treatment (Figure 2A,B).

### 1.2 mEHT-Induced Capillary Damage Accompanied by Tumor Tissue Hypoxia

**Figure 3:**
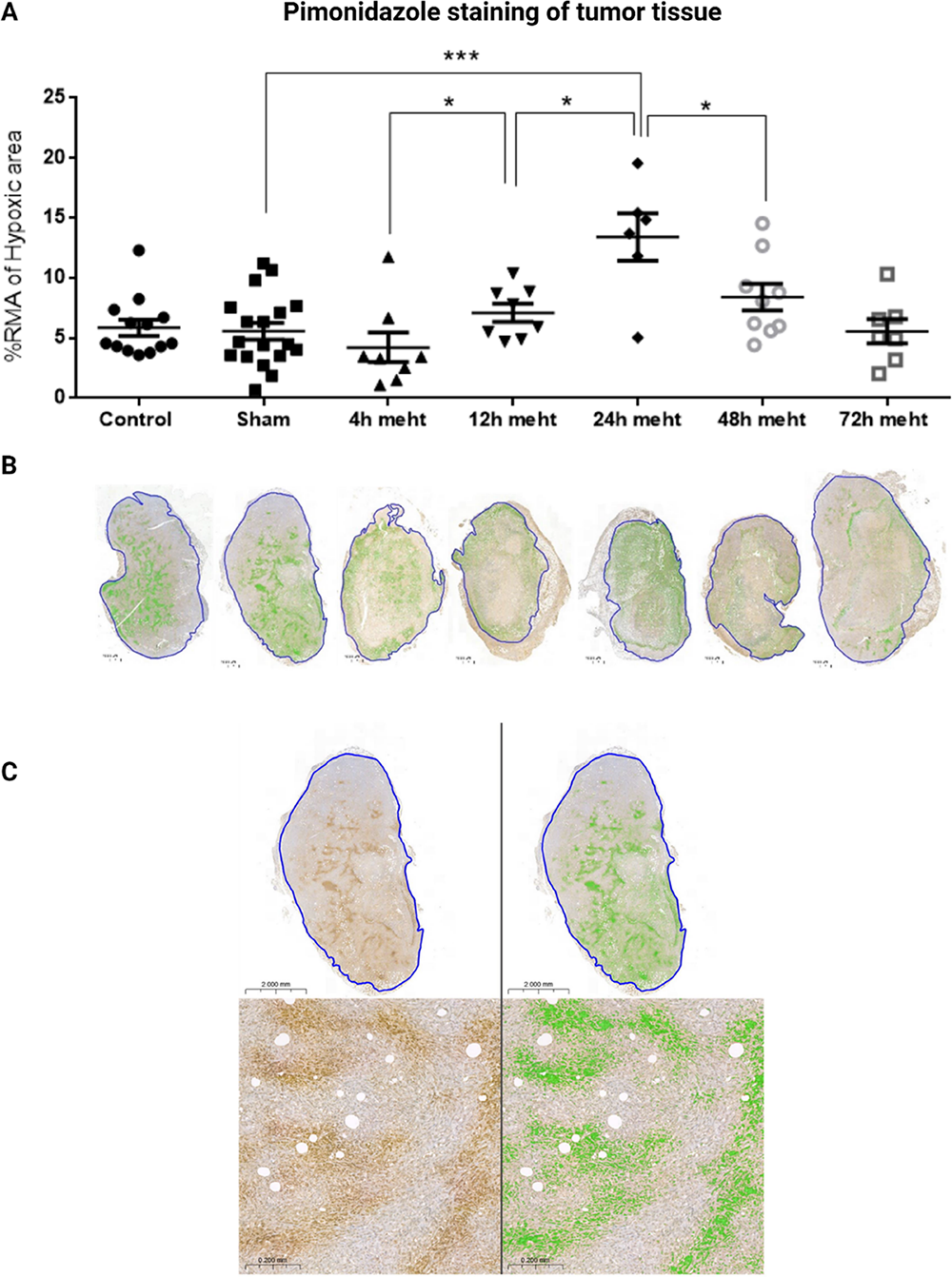
Kinetics of pimonidazole (hypoxia) staining of tumor tissues. (A) Quantification of relative pimonidazole-positive area (green masked area within the blue annotated tumor). (B) Representative images of stained tumor samples with pimonidazole-positive area masked green (0.8×). (C) Representative images for specific staining and masking of pimonidazole stain in histology samples (1×, 10×) RMA: relative mask area. Unpaired Mann–Whitney test. Mean ± SEM, *n* = 6–12/group, *p < 0.05, ***p < 0.001.

Untreated control and sham-treated tumors had similar relative mask areas of pimonidazole staining (specific: dark brown/DAB/) per tumor area of the whole tumor cross section (Figure 3C). Pimonidazole staining demonstrated significant hypoxia 4 and 12 h after the last mEHT treatment. Staining peaked at 24 h, followed by a downward trend reaching the sham level by 72 h (Figure 3A).

### 1.3 Angiogenic Repair Initiated in Response to mEHT Treatment Induced Hypoxia

**Figure 4:**
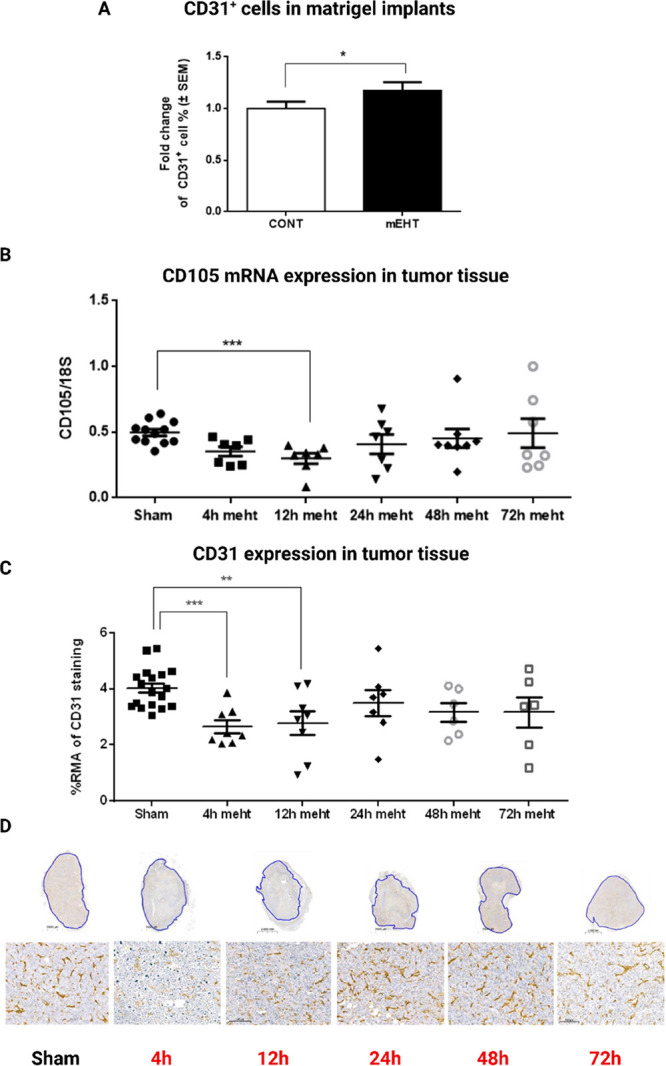
mEHT induced diminution of CD31+ and CD105+ cells recovered by 24 h. (A) Enrichment of CD31+ (blood endothelial cell marker) cells in matrigel in response to mEHT after 24 h. (B) mRNA expression of CD105 (endoglin: tumor vasculature marker). (C) Quantification of relative CD-31 staining in tumors. (D) Representative images of stained tumor samples with CD-31-stained area masked green (0.8×, 15×); RMA: relative mask area (green within the blue annotated area on (D). (A) Paired *t* test, *p < 0.05. (B) Unpaired Mann–Whitney test. Mean ± SEM, *n* = 6–12/group, ***p = 0.0002. (C) Unpaired Mann–Whitney test. Mean ± SEM, *n* = 6–18/group, **p = 0.004, ***p = 0.0002.

In pure matrigel implants not containing cancer cells, mEHT treatment resulted in a significant increase of CD45-CD31+ (endothelial) cells as compared to the sham-treated matrigel (Figure 4A). We also observed significant CD-105 downregulation 12 h after mEHT treatment, with gradual recovery thereafter (Figure 4B). In addition, CD-31 immunohistochemical staining of resected tumor tissues demonstrated a significant reduction of CD31 expression at 4 and 12 h post mEHT, followed by the recovery of CD31 expression at 248–72 h after the last mEHT treatment (Figure 4C,D).

### 1.4 Digoxin Enhanced the Tumor-Killing Effect of mEHT In Vivo

**Figure 5:**
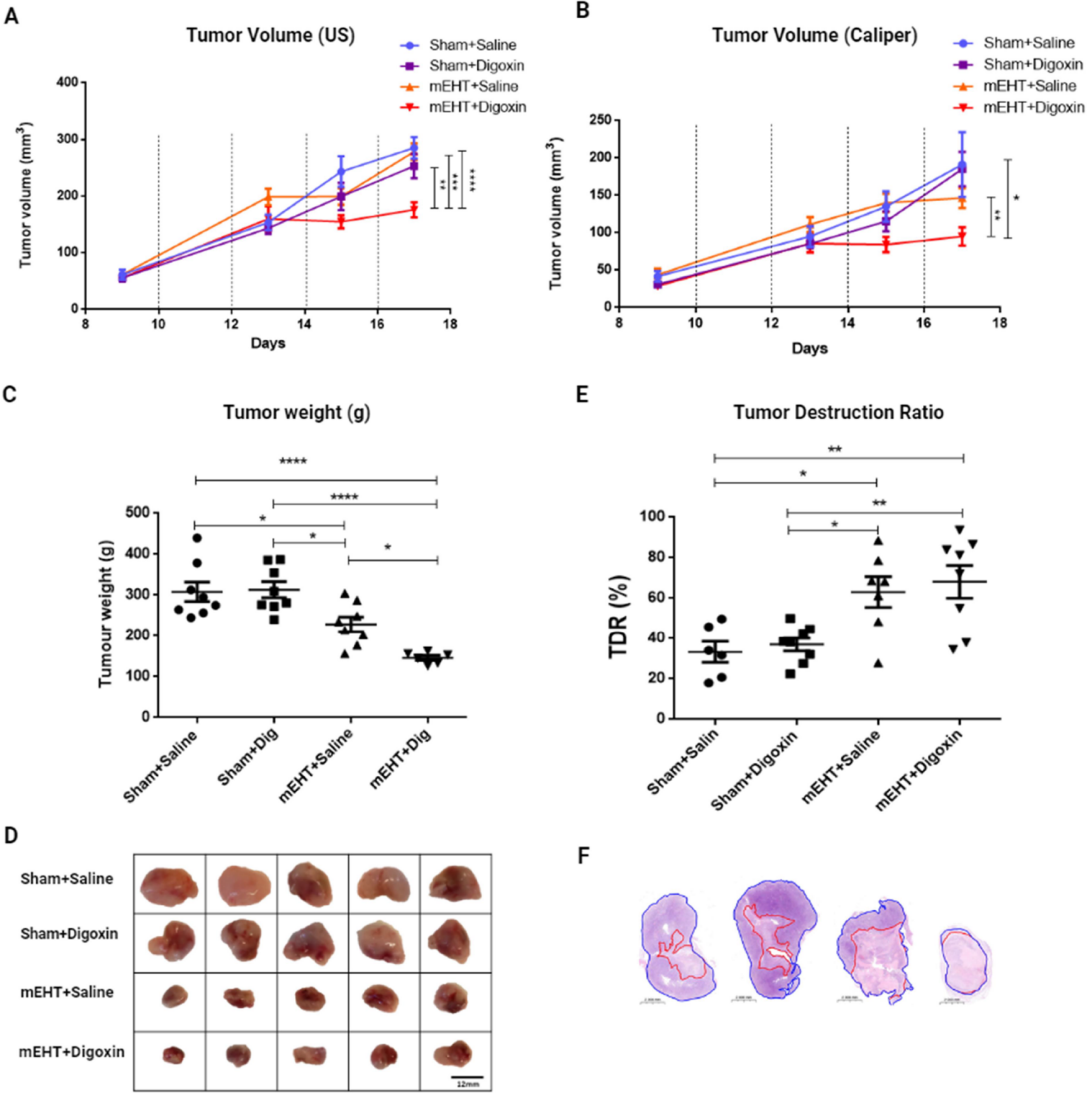
Digoxin provided synergistic tumor growth inhibitory effects when combined with mEHT. (A, B) Tumor volume monitoring by ultrasound (A) and digital calipers (B) after four treatments (dotted lines). (C) Tumor weight. (D) Representative scale images of the excised tumors. (E) TDR: Tumor destruction ratio. (F) Representative images of tumor tissue histology: (A, B) two-way ANOVA, Tukey correction, *n* = 6–8/group; (A) **p < 0.005, ***p = 0.001, ****p = <0.0001; (B) *p < 0.05, **p = 0.005. (C) One-way ANOVA, Tukey correction, *n* = 6–8/group, *p < 0.05, ****p < 0.0001. (D) Scale bar = 12 mm. (E) One-way ANOVA, Tukey correction, *n* = 6–8/group, *p < 0.05, **p < 0.008.

Tumor volume increased constantly in sham+saline-treated mice. Digoxin monotherapy did not influence tumor volume (Figure 5A,B) during the study or tumor weight (Figure 5C) at the end of the study compared to sham-treated tumors (Figure 5A,B). Although the effects of mEHT monotherapy were not reflected in the tumor size (Figure 5A,B), mEHT alone did reduce the tumor weight significantly as compared to sham at the end of the study (Figure 5C). The combined mEHT and digoxin treatment had a significant synergistic effect on tumor weight reduction not only as compared to sham and digoxin monotherapy but also as compared to mEHT monotherapy (Figure 5C,D). Correspondingly, digoxin alone could not induce significantly different tumor tissue damage as compared to sham. However, mEHT alone and in combination with digoxin treatment induced stronger tumor tissue damage compared to sham and digoxin monotherapy. Even though there was a slight difference, the combination therapy did not result in a significantly different tumor tissue damage as compared to mEHT monotherapy (Figure 5E,F).

### 1.5 Digoxin Reduced Tissue Hypoxia Signaling: HIF1-α Expression in mEHT-Treated Tumors

**Figure 6:**
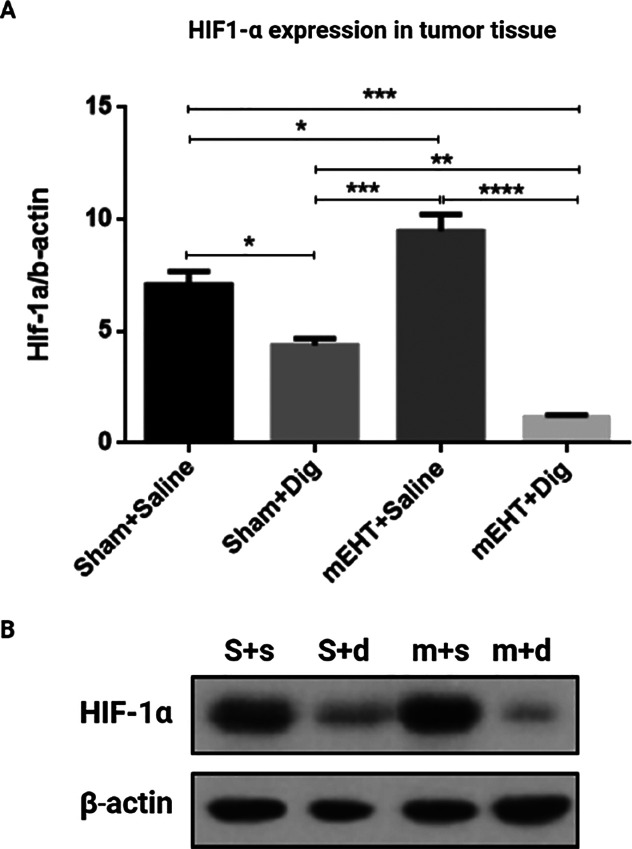
Digoxin reduced tumor hypoxia and angiogenesis when combined with mEHT. (A) Relative fold change of Hif1-α expression as compared to b-actin. (B) Representative images of Western blot (S + s = Sham + saline, S + d = Sham + digoxin, m + s = mEHT + saline, m + d = mEHT + digoxin). (A) One-way ANOVA, Tukey correction, *n* = 6–8/group, *p < 0.05, **p = 0.008, ***p < 0.0005, ****p < 0.0001.

Digoxin alone reduced Hif1-α expression as compared to sham+saline-treated tumors. Hif1-α expression increased significantly in mEHT-treated tumors as compared to sham+saline and digoxin monotherapy. Combined mEHT+digoxin therapy strongly and significantly reduced Hif1-α expression in contrast to sham, digoxin monotherapy, and mEHT monotherapy (Figure 6A,B).

### 1.6 Digoxin Inhibited Blood Vessel Density in mEHT-Treated Tumors

**Figure 7:**
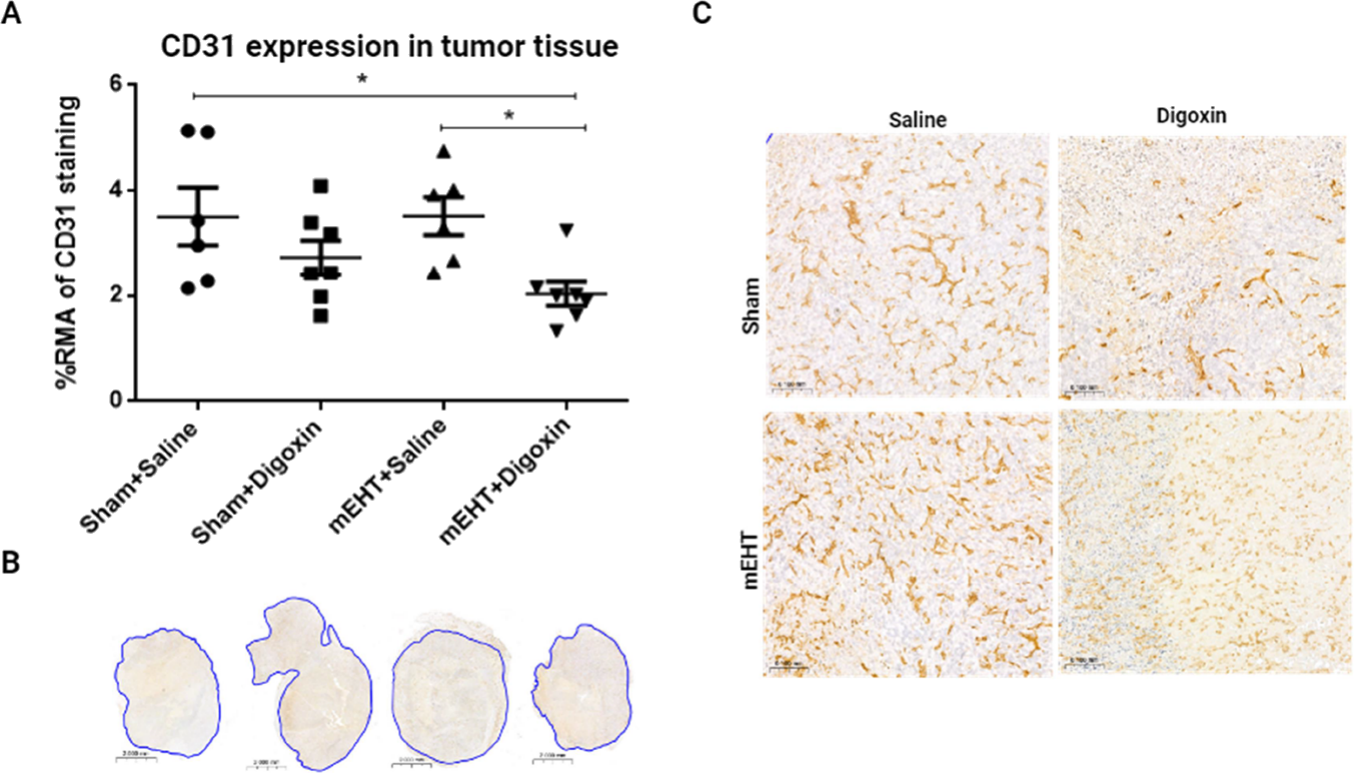
Digoxin reduced vascular density in mEHT-treated tumors. (A) Quantification of relative CD-31 staining in tumors. (B, C) Representative images of CD-31-stained tumor samples (B = 0.8×, C = 15×). (A) One-way ANOVA, Tukey correction, *n* = 6–8/group, *p < 0.05.

After 24 h of the last mEHT treatment, CD-31 expression in both sham and mEHT-treated tumors was similar to that observed before. Similarly, digoxin alone could not significantly inhibit the angiogenic marker expression. However, CD-31 expression was significantly reduced in the combined mEHT + digoxin therapy as compared to mEHT monotherapy (Figure 7A,C).

## 2 Discussion

Our paper is the first study that investigated tumor hypoxia and related angiogenic repair in cancers subjected to repeated mEHT treatments over a shorter span of time. Owing to the heterogeneous nature of tumor tissue, tumor vasculature is a highly complex mediator of tumor proliferation and dissemination.^35,36^ It is also an important determinant to maximize the effects of therapeutic interventions like radiotherapy and chemotherapy.^37^ Conventional/whole body hyperthermia (HT) promotes vasodilation and hence increases blood flow to the tumor, which can on one hand facilitate radiation and chemotherapy perfusion while on the other hand promote tumor growth by providing nutrients and a dissemination route to the tumor cells.^38^

Principally, in mEHT, the local heating of the tumor tissue is achieved by energy absorption, while the blood flowing through the tumor at a physiological temperature acts as a cooling agent. Interestingly in the case of loco-regional hyperthermia such as mEHT, the energy absorption, i.e., heating and the dissipation by flowing blood, is independent. Therefore, a dynamic balance yet a constant small temperature gradient results in heterogenic heating that represents the heterogeneous tumor vasculature.^26^ This heterogeneous heating therefore provides better cytotoxic effects.

In clinical settings, patients received multiple mEHT treatments, i.e., an average of 28–30 sessions during the treatment.^40,41^ In a clinical study, mEHT treatment three times a week in combination with concurrent chemotherapy provided a better therapy response and increased disease-free survival in patients with lymph-node metastasis in locally advanced cervical cancer.^40^ Similarly, mEHT in combination with chemotherapy provided a better overall survival in cervical cancer patients.^41^ Since the mEHT dosage depends on the specific energy absorbed over the course of treatment,^39^ the minor temperature difference between the tumor tissue and its vasculature can accumulatively be of significant clinical value. It was therefore clinically relevant to investigate how tumor vasculature responds to repeated mEHT treatments.

Tumor vascularization and oxygenation responses to hyperthermia are variable and complex. Effects of HT on blood flow and vascular damage have been extensively researched.^42^ However, the effect of mEHT on the blood flow and vascular damage still needs investigation. However, mild HT has been reported to promote tumor reoxygenation.^43−46^ Other studies report a reduction in tumor oxygenation due to vascular damage^45^ or transient reoxygenation.^47^ Furthermore, the relationship between blood perfusion and oxygenation of the tumor tissue is not linear 24 h after HT.^48^ Interestingly, tumors subjected to HT for a cumulatively higher time/dosage experience hypoxia and vascular damage, which is in line with our findings (Figures 3 and 4).^44,49^ Furthermore, we also observed vascular recovery in our cancer model after 24 h (Figures 1 and 4). Our results demonstrate that hypoxia peaked at 24 h after mEHT (Figure 4) as compared to sham tumors, which is in line with previous studies.^50^ However, these findings are different from that of Kim et al., who compared the effects of mEHT and conventional HT. They observed a small reduction in hypoxia after mEHT,^51^ which could be due to two reasons. First, Kim et al. treated tumors with mEHT only once, while we did multiple mEHT treatments. Second, the tumors were harvested 72 not 24 h after mEHT by the former group. In line with the findings of Kim et al., we observed reduction in hypoxia at the 72 h time point as well.

**Figure 8:**
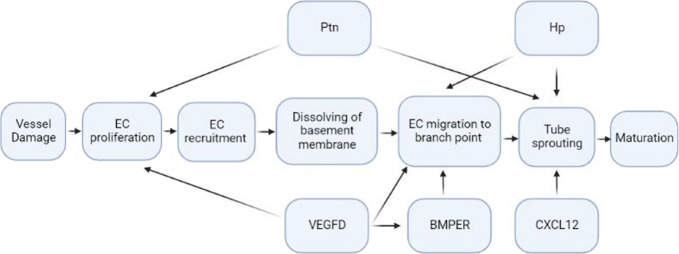
Theoretical network of hypoxia-responsive genes modulating angiogenesis.

As it is already reported that mEHT elicits stress responses in cancer,^52−58^ we hypothesized that tissue hypoxia triggers another stress response which is translated to angiogenic repair. A significant upregulation in CD-31^+^ and CD-45^–^ endothelial cells 24 h after the last mEHT treatment in matrigel implants strengthened our hypothesis. Furthermore, our time kinetics study demonstrated a reduction in the expression of vascular endothelial markers, CD105 and CD-31, after 12 h, followed by recovery in expression after 24 h of mEHT treatment, which endorses our hypothesis. Additionally, in a multiplex analysis of mEHT-treated tumors,^52^ we found previously a number of genes including *Hap*toglobin (*Hp*), *V*ascular *E*ndothelial *G*rowth *F*actor-*D* (*VEGF-D*), *P*leio*t*ropi*n* (*Ptn*), *BMP*-binding *E*ndothelial *R*egulator (*BMPER*), and chemokine (*C-X-C* motif) *l*igand-12 (*CXCL-12*) with a significantly high overexpression. Roles of these genes as angiogenic activators in response to hypoxic stress have already been established in brain, cardiac, kidney, and cancer cells.^59−68^ We therefore believe that these genes facilitate angiogenesis by modulating its one or more steps (Figure 8 and Table 1).

**Table 1:** Cellular Stress Response Related Genes, Upregulated by mEHT Treatment, as Detected by Different Multiplex Assays (Next-Generation Sequencing (NGS), Nanostring, and Mass Spectrometry (MS)^a^

Nr.	gene name	description	NGS		nanostring	
	abbreviation	full name	mRNA			
statistics			FC	*p*	FC	*p*
1	*Bmper*	BMP-binding endothelial regulator	15.65	0.00042	no DE	
2	*Hp*	haptoglobin	9.7	0.0002	7.5	2.5 × 10^–04^
3	*VEGFD*	vascular endothelial growth factor D	9.9	0.0030	15.7	2.59 × 10^–05^
4	*Ptn*	pleiotropin	7.8	0.0099	7.6	8.2 × 10^–04^
5	*CXCL12*	chemokine (C-X-C motif) ligand 12	6.80	0.0016	no DE	

a Genes (official name abbreviations are used in all multiplex platforms and descriptions of the coded proteins) detected as upregulated. Cell line: 4T1, 3x mEHT-treated. (DE, differential expression).

We aimed to inhibit hypoxia signaling with the cardiac glycoside digoxin. It is noteworthy that in the literature digoxin had been administered for 14–40 days at a concentration of 2 mg/kg daily and needed an average of 21 days of treatment alone or in combination with other chemotherapeutics to observe significant tumor volume reduction. Gayed et al., however, administered digoxin only for 7 days and could observe significant reduction of Hif1-α and CD-31 expression, but they could not describe any significant reduction in tumor growth over this short treatment period.^69^ We, for the first time, observed significant tumor weight reduction by day 8 of digoxin administration when combined with mEHT treatment; hence, mEHT accelerated the anticancer effects of digoxin. Furthermore, we observed a reduction in Hif1-α expression and hence hypoxia signaling and inhibition of angiogenesis in tumors subjected to combination therapy.

The data are of valuable importance as the results have dual interpretations. Our data explicate a collaborative relationship between mEHT and digoxin. The combination can provide better outcomes by mEHT enhancing digoxin functionality, reducing tumor weight over a shorter span of time, and digoxin reducing the hypoxic and angiogenic response initiated by repeated mEHT. Further research on downstream targets of Hif1-α modulated in response to combined digoxin and mEHT would be of valuable importance but was beyond the scope of this paper. In conclusion, our findings corroborate the efficiency of mEHT as both a complementary treatment and monotherapy in our TNBC model. We report for the first time an elaborate time-related vascular and hypoxic response to repeated mEHT treatments. Furthermore, our findings substantiate the role of digoxin as an anticancer therapy that could reduce hypoxia, angiogenesis, and tumor growth over a shorter treatment regimen when combined with mEHT. We believe that combining mEHT with digoxin can provide better results over a shorter span of time in clinical settings.

## 3 Materials and Methods

### 3.1 Cell Culture

4T07 and 4T1 cells were grown as adherent culture in Dulbecco’s modified essential medium (DMEM, 4.5 g/L glucose with l-glutamine, #12-604F, Lonza A. G., Basel, Switzerland) supplemented with 10% fetal bovine serum (FBS Catalogue#ECS0180L, Euroclone S.p.A., Pero, Italy) and 10% penicillin–streptomycin (#17-602E, Lonza A. G., Basel, Switzerland).

### 3.2 In Vivo Model

Six to eight weeks' old female BALB/c mice were raised in the SPF animal facility of the Department of Oncology, Semmelweis University with ad libitum access to standard rodent chow and water, under 12 h dark/12 h light cycles. Animals were anesthetized for tumor cell inoculation with isoflurane (Baxter International Inc., Deerfield, IL, USA) in 4–5% concentration for induction and 1.5–2% to maintain anesthesia with 0.4–0.6 L/min compressed airflow. 1 × 10^6^ 4T1 cells in 50 μL phosphate-buffered saline (PBS; without calcium and magnesium #17-516F, Lonza A.G., Basel, Switzerland) solution were subcutaneously inoculated by 50 μL Hamilton syringe (Hamilton Company, Reno, NV, USA). Inoculation was made orthotopically into the fourth mammary gland’s fat pad in each mouse. Eight days after inoculation, tumors were measured with a digital caliper and ultrasound, and mice were randomized into mEHT and sham-treated groups according to the tumor size and body weight. Mice were injected daily with 2 mg/kg dose of digoxin (Merck Life Science Kft. D6003) or saline for 8 days. Twenty-four hours after the last treatment, mice were euthanized by cervical dislocation under anesthesia. The tumors were resected and cleaned of the surrounding connective tissue, fat, and skin. The condition of the internal organs (bowels, urinary bladder, and spleen) and possible adherence between the tumor and muscles were inspected. Tumors were cut in half along their longest diameter, and one-half was placed in a 4% buffered formaldehyde solution (Molar Chemicals Kft., Halásztelek, Hungary). The other half of the tumors was frozen in liquid nitrogen for molecular analysis (RNA isolation, RT-PCR). For time kinetics experiments, tumors were harvested at different time points: 4, 12, 24, 48, and 72 h after the last treatment, and mice were treated with pimonidazole hydrochloride (hypoxyprobe1, HPI catalogue no. HP1-200) by tail vain injection 1 h before tumor harvest. Interventions and housing of the animals conform to the Hungarian Law Nos. XXVIII/1998 and LXVII/2002 about the protection and welfare of animals and the directives of the European Union. The animal experimental protocol was approved by the National Scientific Ethical Committee on Animal Experimentation under Nos. PE/EA/633-5/2018 and PE/EA/50-2/2019.

### 3.3 In Vivo mEHT Treatments

Tumors were treated three to five times with the newly developed rodent-modulated electrohyperthermia device, as described previously.^52,53^ The principle of the treatment is a capacitive-coupled, amplitude-modulated, 13.56 MHz electromagnetic field that transfers energy to the tumors. Animals were placed on a heating pad (in vivo applicator), functioning as the lower electrode, and connected to a LabEHY-modulated electro hyperthermia 200 device with heating and a radiofrequency (RF) cable. The abdominal area below the mobile electrode and the back of the mice were shaved before the treatments to enable electric coupling. Treatments were performed with a LabEHY 200 device in a temperature-driven way for 30 min with 0.7 ± 0.3 W after a 5 min long warmup. Temperature monitoring was performed with an optical temperature sensor Luxtron (Oncotherm Ltd., Budaörs, Hungary). Temperature parameters were set and monitored as per our previously demonstrated guidelines.^39^ During sham treatments, the electromagnetic field was turned off, but all other conditions (heat pad temperature and upper electrode position) were similar to the mEHT treatment.

### 3.4 Matrigel Plug Assay and Flow Cytometry

To visualize and analyze the effect of mEHT on vascularization in vivo, we performed a Matrigel plug assay. Eight to ten weeks old male C57BL/6 mice were raised in the Department of the Animal Facility of the Basic Medical Science Center of Semmelweis University. 500 μL of liquid Matrigel (BD Biosciences) containing 600 ng/mL bFGF was injected subcutaneously into the left and right groin regions of mice. On day 3, the right-side plugs were treated with mEHT, and the left-side plugs were used as controls. The treatment was repeated twice every other day. After 8 days, the plugs were excised and subjected to measure the hemoglobin content by a hemoglobin assay kit (Sigma-Aldrich) or to flow cytometry.

Matrigel plugs were treated with Liberase TM (Roche Diagnostics) at 37 °C for 30 min. The digested plugs were then filtered through a 70 μm cell strainer, and red blood cells were eliminated by Red Blood Cell Lysis buffer (BioLegend; San Diego, CA, USA), centrifuged for 5 min at 350 × *g*, washed with PBS, and fixed and permeabilized with an Intracellular Fixation and Permeabilization Set (eBioscience). TruStain FcX antibody (Biolegend; San Diego, CA, USA) was used for blocking the nonspecific binding of IgG to the Fc receptors. Immunostaining was performed by incubating the cells with monoclonal antibodies for 30 min on ice. The following antibodies were used: PE antimouse CD31 antibody and APC antimouse CD45 antibody (Table 2; Biolegend, San Diego, CA, USA). Flow cytometry was performed with a FACS Calibur (Becton Dickinson, Mountain View, CA, USA). Frequency and intensity measurements were calculated in CellQuest software (Beckton Dickinson).

**Table 2:** Antibodies Used for Flow Cytometry

antibody	type	catalogue no.	vendor
*APC CD-45*	Mouse, mAb	103111	Biolegend
*CD-31*	Mouse, mAb	102407	Biolegend

### 3.5 qPCR

RNA isolation was performed with TRI reagent (Molecular Research Center LLC, Ohio, USA) according to the manufacturer’s instructions. Isolated RNA was reverse-transcribed by a High-Capacity cDNA Reverse Transcription Kit (Applied Biosystems, Carlsbad, CA, USA). The amplified cDNA was used as a template for RT-PCR. Messenger RNAs were detected in the samples by SYBR Green-based RT-PCR with SsoAdvanced Universal SYBER Green Supermix and the CFX96 Touch Real-Time PCR Detection System (Bio Rad, Hercules, CA, USA). Expressions were normalized to 18S. The used primers are listed in Table 3.

**Table 3:** Primers Used for RT-PCR

gene symbol	gene name	primer pairs
*18S*	18S [*Mus musculus*]	Fwd: CTCAACACGGGAAACCTCACRev: CGCTCCACCAACTAAGAACG
*CD105*	endoglin [*Mus musculus*]	Fwd: TGGATACCGGATAAGGCCCARev: CCGACTCTTTCTGCGAGACC

### 3.6 Histology and Immunohistochemistry

Formalin-fixed tumor samples were dehydrated and embedded in paraffin. Serial sections (2.5 μm) were cut and mounted on salinized glass slides and kept in a thermostat at 65 °C for 1 h. Sections were dewaxed and rehydrated for H&E staining and immunohistochemistry (IHC). Endogenous peroxidases were blocked for 15 min using 3% H_2_O_2_ in methanol. For antigen retrieval, the slides were subjected to constant heating for 20 min in Tris–EDTA (TE) buffer pH 9.0 (0.1 M Tris base and 0.01 M EDTA) or citrate buffer pH 6.0 (Dako, Glostrup, Denmark)) for CD-31 and antipimonidazole staining, respectively, using an Avair electric pressure cooker (ELLA 6 LUX(D6K2A, Bitalon Kft, Pécs, Hungary), followed by a 20 min cooling with an open lid. The nonspecific proteins were blocked by incubation with 3% bovine serum albumin (BSA, #82-100-6, Millipore, Kankakee, Illinois, USA) diluted in 0.1 M Tris-buffered saline (TBS, pH 7.4) containing 0.01% sodium azide for 20 min. The sections were incubated, with the primary antibodies diluted in 1% BSA/TBS + TWEEN (TBST, pH 7.4) (Table 1) overnight in a humidity chamber. Peroxidase-conjugated antirabbit and antimouse IgGs (HISTOLS-MR-T, micropolymer – 30011.500T, Histopathology Ltd., Pécs, Hungary) were used for 40 min incubations, and the enzyme activity was revealed in a 3,3′-diaminobenzidine (DAB) chromogen/hydrogen peroxide kit (DAB Quanto-TA-060-QHDX-Thermo Fischer Scientific, Waltham, MA, USA) under microscopic control. All incubations were performed at room temperature, with the samples washed between incubations in TBST buffer for 3 × 3 min. Digital evaluation of the tumor destruction ratio (TDR%) on H&E slides and the CD31 and pimonidazole staining was performed using the CaseViewer software as described earlier (Table 4).^39^ The red blood cell-covered area was quantified by the CaseViewer software; The RBC-covered area was masked relative to the whole annotated tumor area by double masking. The number of viable blood capillaries per tumor area was manually counted, and viable vessels were characterized as circular, luminal, surrounded by living cells, visibly containing RBCs with area no less than 100 μm^2^.

**Table 4:** Antibodies Used for Immunohistochemistry

antibody	type	catalogue no.	dilution	vendor
anti-pimonidazole	mouse, mAb	4.3.11.3	1:50	hypoxyprobe
CD-31	rabbit, mAb	77699S	1:100	cell signaling

### 3.7 Western Blot Analysis for HIF-1α

Total protein isolation was performed with TRI reagent (Molecular Research Center lnc., Ohio, USA) according to the manufacturer’s instructions. 20 μg of protein was loaded per well and fractionated on 12% SDS-PAGE gel and transferred to a PVDF membrane. The membrane was cut to two in order to probe the same membrane for two proteins simultaneously. The membranes were probed with a primary antibody specific for HIF-1α and β-actin overnight at 4 °C (Table 5). The membrane was then incubated with an HRP-conjugated secondary antibody for an hour. Chemiluminescent signal was detected by SuperSignal West Pico PLUS Chemiluminescent Substrate (ThermoFisher Scientific, catalogue # 34578). The chemiluminescent signal was detected by an X-ray film, and the blot was analyzed by ImageJ software.

**Table 5:** Antibodies Used for the Western Blot^a^

antibody	type	catalogue no.	dilution	vendor
HIF-1α	mouse, mAb	sc-13515	1:200	Santa Cruz Inc.
β-actin	mouse, mAb	ab6276	1:5000	Abcam
Anti-mouse IgG		7076	1:3000	cell signaling

a HIF-1α: hypoxia-inducible factor 1-alpha; IgG: immunoglobulin G; mAb: monoclonal antibody.

### 3.8 Statistical Analysis

Statistical analysis was done using the GraphPad Prism software (v.6.01; GraphPad Software, Inc., La Jolla, CA, USA). Unpaired Mann–Whitney nonparametric tests were performed in the comparison of sham and mEHT-treated groups. Follow-up examinations for more than two groups were statistically evaluated with two-way ANOVA or one-way ANOVA with Tukey correction. Differences were considered statistically significant as *p < 0.05, **p < 0.01, ***p < 0.001. Data are presented as mean ± SEM.

## Data Availability

All data associated with this study are presented in the paper. The data that support the findings of this study are available from the corresponding author upon reasonable request.

## Notes

The studies did not involve human participants, human data, or human tissue. Interventions and housing of the animals conformed to the Hungarian Law Nos. XXVIII/1998 and LXVII/2002 about the protection and welfare of animals and the directives of the European Union. All animal procedures were approved by the National Scientific Ethical Committee on Animal Experimentation under No. PE/EA/50-2/2019.

## Notes

The manuscript does not contain any individual person’s data in any form (including individual details, images or videos).

## Notes

We did not use materials from external sources to prepare our manuscript.
