# Correction to “Digoxin-Mediated Inhibition of Potential Hypoxia-Related Angiogenic Repair in Modulated Electro-Hyperthermia (mEHT)-Treated Murine Triple-Negative Breast Cancer Model”

**DOI:** 10.1021/acsptsci.4c00094

**Published:** 2024-02-28

**Authors:** Syeda Mahak Zahra Bokhari, Kenan Aloss, Pedro Henrique Leroy Viana, Csaba András Schvarcz, Balázs Besztercei, Nino Giunashvili, Dániel Bócsi, Zoltán Koós, Andrea Balogh, Zoltán Benyó, Péter Hamar

Page 460. In Figure 5C of our article, the tumor weight was mistakenly given in grams instead of milligrams. The corrected figure panel is shown below:

**Figure 5:**
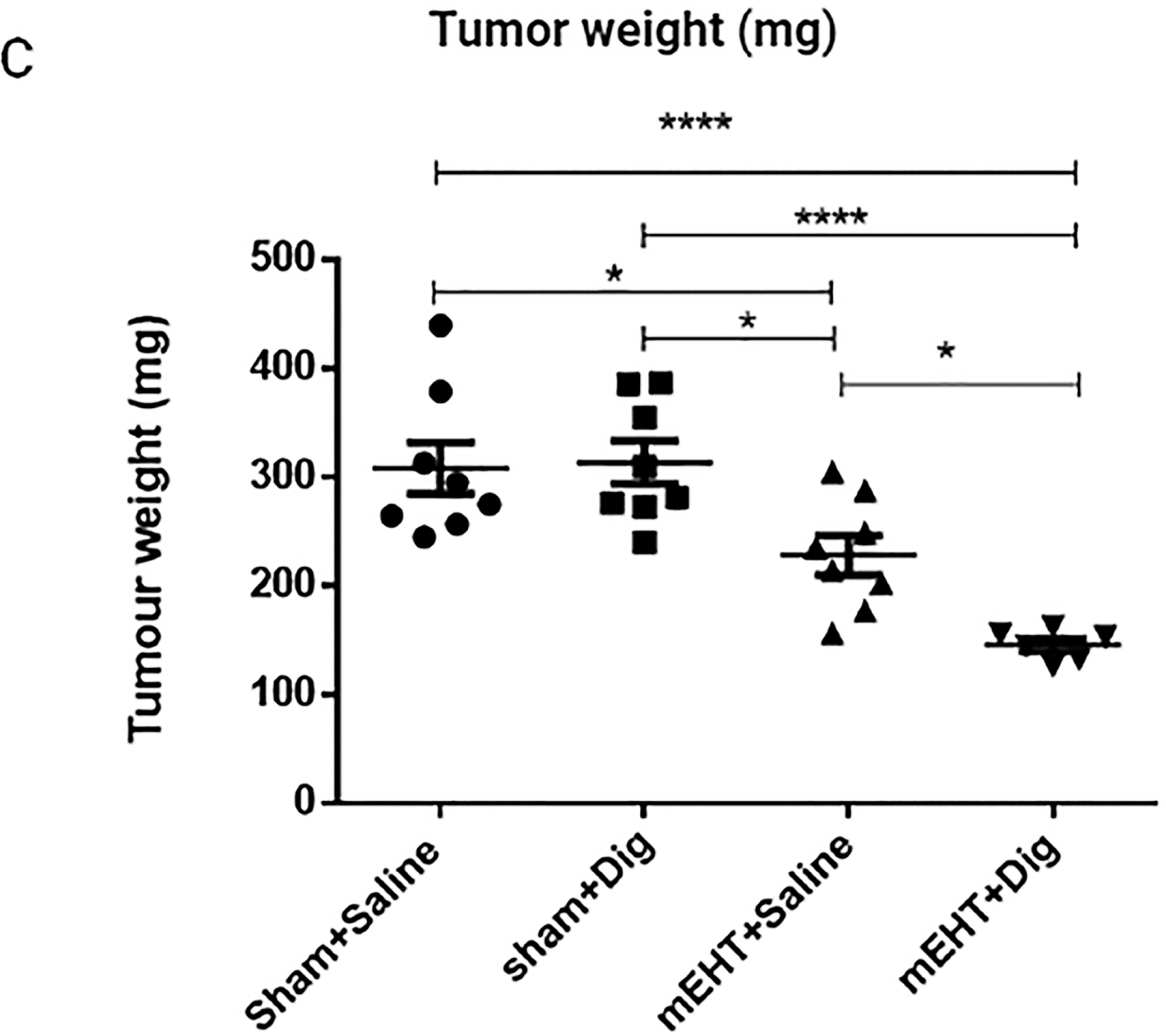
Digoxin provided synergistic tumor growth inhibitory effects when combined with mEHT. (C) Tumor weight. One-way ANOVA, Tukey correction, *n* = 6–8/group, *p < 0.05, ****p < 0.0001.

Page 464. The affiliation “Cerebrovascular and Neurocognitive Disorders Research Group”, associated with author Csaba András Schvarcz in the original article, must be corrected and linked additionally to author Zoltán Benyó. Their Author Information should now read as follows:

**Csaba András Schvarcz** – *Institute of Translational Medicine, Semmelweis University, Budapest 1085, Hungary; HUN-REN-SU Cerebrovascular and Neurocognitive Diseases Research Group, Budapest 1094, Hungary*

**Zoltán Benyó** – *Institute of Translational Medicine, Semmelweis University, Budapest 1085, Hungary; HUN-REN-SU Cerebrovascular and Neurocognitive Diseases Research Group, Budapest 1094, Hungary*

